# How to Facilitate Adherence to Cardiac Rehabilitation in Primary Health Settings for Ischaemic Heart Disease Patients. The Perspectives of Health Professionals

**DOI:** 10.3389/fresc.2022.837175

**Published:** 2022-03-28

**Authors:** Maiken Bay Ravn, Maria Uhd, Marie Louise Svendsen, Lisbeth Ørtenblad, Thomas Maribo

**Affiliations:** ^1^Department of Public Health, Centre for Rehabilitation Research, Aarhus University, Aarhus, Denmark; ^2^DEFACTUM, Central Denmark Region, Aarhus, Denmark

**Keywords:** cardiac rehabilitation, qualitative study, focus groups, dropout, ischaemic heart disease, primary health settings

## Abstract

**Background:**

Cardiac rehabilitation (CR) is a class 1A recommendation and an integrated part of standard treatment for patients with cardiac disease. In Denmark, CR adheres to European guidelines, it is group-based and partly conducted in primary health settings. Despite high evidence for the benefits of CR, it remains underutilized. How to facilitate CR adherence in primary health settings is poorly understood.

**Aim:**

This study explores health professional's perspectives on how to facilitate CR adherence for patients with ischaemic heart disease in primary health settings.

**Methods:**

Data were collected through focus group discussions. Respondents were health professionals specialized in and working with CR in primary health settings. Data were analyzed using thematic analysis.

**Results:**

Eleven health professionals participated in two focus group discussions. Five themes emerged as facilitators of CR; (1) placing the person at the center, (2) coherent programme, (3) flow of information, (4) contextual factors, and (5) feeling of belonging.

**Conclusion:**

This study illuminates the complexity of facilitating adherence to CR in primary health settings and provides ways in which health professionals may facilitate adherence. Placing the person at the center is pivotal and may be done by adapting CR offers to patients' social context, culture and life circumstances and ensuring a feeling of belonging. The rhetoric related to CR should be positive and throughout the entire course of treatment health professionals should possess a generic and collective approach to and view of CR. Perceiving these elements as potential facilitators is of vital importance and addressing them may facilitate adherence.

## Introduction

Worldwide, ischaemic heart disease (IHD) is the most common cause of death due to non-communicable diseases (1). This underlines the need for efficient cardiac health services. Cardiac rehabilitation (CR) is a class 1A recommendation and an integrated part of standard treatment for patients with cardiac disease (2, 3). CR aims to provide cardiac patients with the best possible conditions mentally, physically and socially in order for them to resume or preserve a normal life (4). CR is a multi-faceted disease management programme that may reduce rehospitalisation, all-cause mortality, cardiac mortality and activity-related symptoms while improving functioning (5–8).

CR includes several different aspects, such as lifestyle (exercise, healthy diet and smoking cessation), screening for anxiety and depression, psychosocial support, patient education and return to work (9, 10). According to European guidelines, CR is group based and partly conducted in primary health settings (9). According to the World Health Organization (WHO), rehabilitation in primary health settings enables patients to remain in education and continue being part of the workforce while reducing rehospitalisation (11). Even though CR has benefits for both patient and society, CR remains underutilized (6, 12, 13). Patient-related characteristics associated with dropout from CR have been identified and include high age, poor exercise capacity, comorbidities and employment status (14). In order to understand how adherence to CR can be facilitated, causes for dropout must be explored. In this study adherence follows quality indicators and benchmark standards used in Denmark (9). However, patients and health professional's perspectives on the causes of dropout are poorly understood. The aim of the present study was to explore health professional's perspectives on how to facilitate CR adherence. Patient's reasons for drop-out were examined in a previous study (15).

## Method

### Setting

Health professionals working with CR in five primary health settings from the same region of Denmark were invited to participate in focus group discussions. The settings covered five municipalities counting a total of 635,000 inhabitants. The settings varied with respect to population size, population density and mix of urban and rural areas and were considered representative. The focus group discussions were guided by two experienced interviewers one MHS and the other RN and MSc in nursing (MBR and MU) and held on an online forum due to the COVID-19 pandemic.

The study complied with the Consolidated Criteria for Reporting Qualitative Research (COREQ) (16).

### Focus Group Participants

The head of the CR team in each primary health setting was contacted and facilitated recruitment. Relevant health professionals in each setting were invited to participate, so that all professions working with CR in their team were represented in the focus groups. Eleven health professionals were invited and participated in two focus group discussions. The health professionals were four nurses, five physiotherapists and two dietitians of mixed age and gender and with a professional experience ranging from 1 to 15 years (mean: 8 years).

### Focus Group Discussion

**Figure 1:**
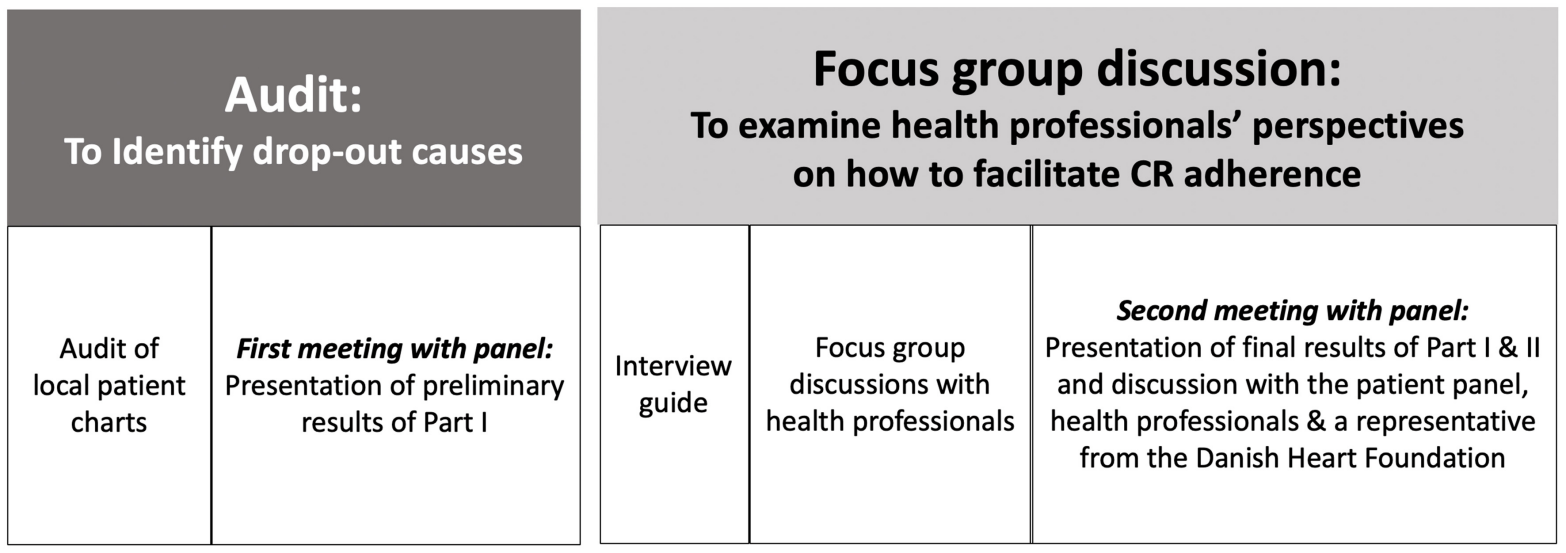
Linkage between studies.

The duration of the focus group discussions was app. 1.5 h. Focus group discussions were initiated with an introduction of all present. A semi-structured interview guide based on results from a qualitative audit of patient's causes for dropout was developed to facilitate discussions (15). An overview of the link between this study and the audit is presented in Figure 1. Furthermore, case presentations of fictive patients were created to represent the audit results, see Supplemental Material. These cases were used to facilitate and qualify the discussion about how to facilitate patient adherence. The participants were informed of the aim and cases beforehand.

### Analysis

The focus group discussions were recorded and transcribed verbatim. NVivo 2.0 software was used to organize and code the data. Furthermore, data were analyzed thematically in a process inspired by the phases presented by Braun and Clark; (1) Reading the transcripts; (2) Generating initial codes; (3) Arranging the codes into themes; (4) Discussing and reviewing the codes and themes; (5) Final analysis and extraction of quotes for analysis (17).

**Figure 2:**
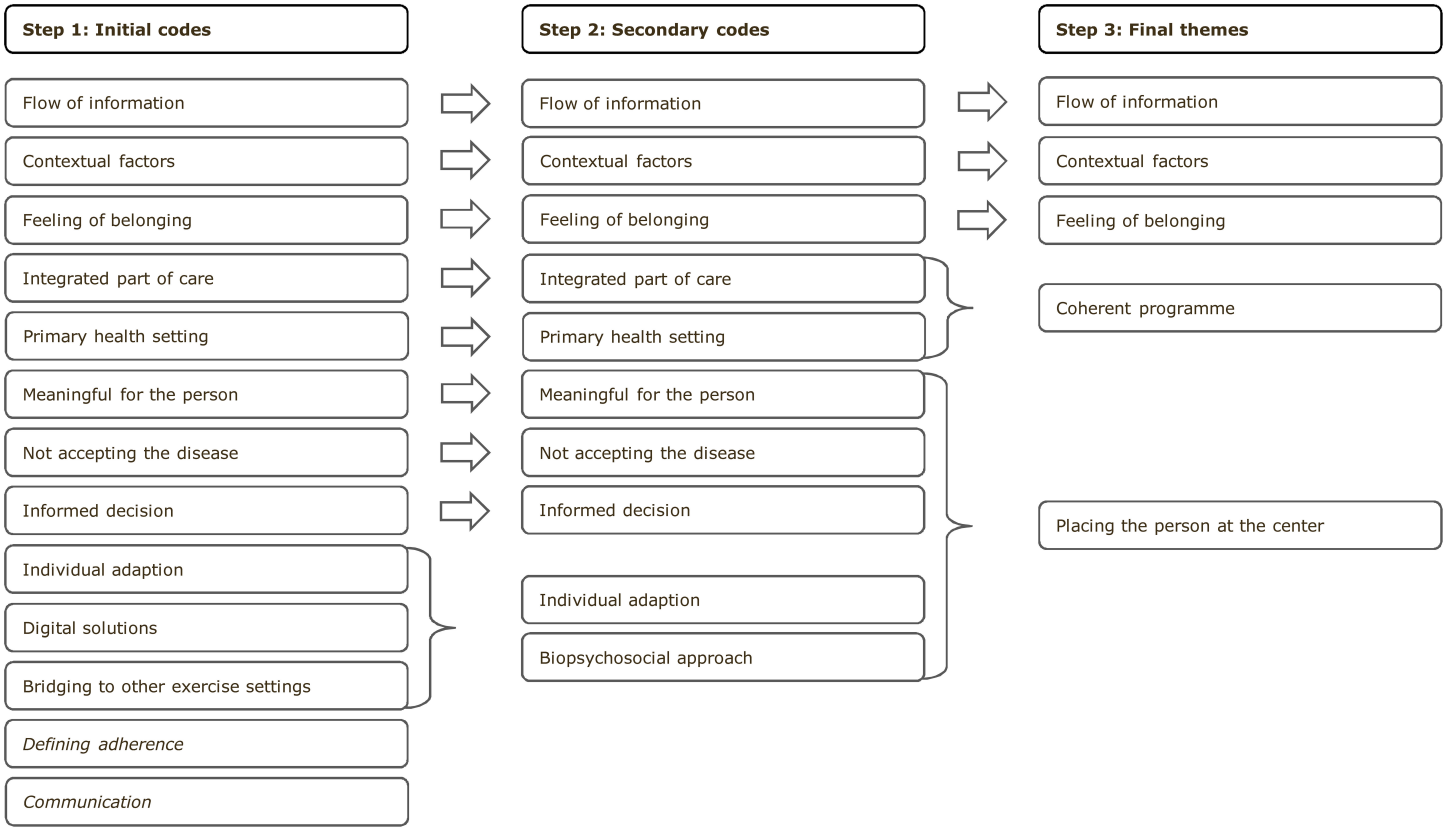
Overview af codes and themes.

An inductive approach was used for coding. Thirteen initial codes were identified and discussed. Agreement was reached to eliminate two codes not related to the aim. Additionally, two codes were renamed and one was added. The transcripts were then revisited and recoded according to the newly defined codes. The codes were discussed and, finally, five themes were identified, see Figure 2. Quotes were selected and extracted to emphasize the results.

### Ethics

The project was approved by the Danish Patient Safety Authority (ID: 31-1522-28). All participants gave written consent to participate. All participants remained anonymous in the data presentation.

### Expert Panel

A panel of former patients with heart disease was formed. Patients were recruited through the Danish Heart Association. Four patients participated. Health professionals from two primary health settings and a representative from the Danish Heart Foundation also participated in the panel. The panel meeting was held online due to the COVID-19 pandemic.

The expert panel was formed to ensure the relevance and transferability of the findings to practice. The results of the focus groups were presented and discussed with the panel. The patients used their own experiences to reflect on the results and deliberate on similarities, differences and ways of addressing barriers. The health professionals and representative from the Danish Heart Foundation provided organizational and governmental perspectives to the discussion. The results of the discussion with the expert panel are presented in the Discussion section and referred to as “Panel.”

## Results

Five themes were identified; placing the person at the center, coherent programme, flow of information, contextual factors, and feeling of belonging.

### Placing the Person at the Center

According to the WHO, rehabilitation is person-centered and should be adapted to individual preferences, needs and goals (18). Person-centered rehabilitation involves focusing on patient autonomy and empowerment, recognizing the role of relatives and putting the person at the center of rehabilitation policy, service delivery and practice (19). In the focus groups, health professionals presented various examples of how they used this approach. Identifying patient's individual barriers through a biopsychosocial approach is one example.

“*What are your challenges and how may we help you?” (nurse)*.

Barriers to adherence may vary, so engaging in a dialogue with each patient and thereby gaining insight into their preferences and challenges may potentially facilitate adherence. Some patients need more support and motivation, whereas others need less. No one size fits all, as illustrated by this quote:

“*I can tell you how we would motivate in this case, but in a million other cases just like this we would do something different, so it all depends on individual competences.” (physiotherapist)*.

Individual adaption of the programme varies from providing individual sessions with patients who find it challenging to engage in large groups to tailoring their exercise needs to their individual fitness capacity.

“*Again, it is all about acknowledging them for attending and individually try to adjust the exercise. I think that the physiotherapists are doing their job impeccably; well, this training exercise is not for you because you have that issue, but you can make this one instead. So, it's all about evaluating; and if the patient feels discomfort, we'll make adjustments for next time.” (nurse)*.

This quote illustrates how the health professionals have already adapted their practice to keeping the person at the center of rehabilitation. In the health professional's perspectives, it was not possible to obtain 100% CR adherence. However, they emphasized the importance of securing that patients make the decision to drop out on an informed basis.

“*It's also about ensuring that we, as health professionals, have given patients enough information to make an informed choice.” (nurse)*.

The health professionals provided examples illustrating how they challenged patient's preconception about CR and why it is an important part of their treatment. Furthermore, in their experience, this occasionally allows the patient to adhere to CR and made them acknowledge that they benefitted more than they initially expected.

### Coherent Programme

Providing CR in primary health settings requires close collaboration with the hospital to ensure a coherent programme. Hospitals are responsible for referring patients to CR; and according to health professionals in the primary health settings, the rhetoric employed about CR during hospitalization plays an important role for adherence to CR.

“*Being part of the treatment is something we can talk with the doctors [at the hospital] about because it is important that the doctors aren't saying: ‘You are going to be fine in 6-8 weeks and then you will be able to do as you normally would'.” (physiotherapist)*.

The importance of both communications internally and across sectors but also the need for a common language and perception on CR across both sectors is according to health professionals needed. Patients are presented with CR in different ways and the health professionals call for a common language and perception of CR from the onset of the treatment. The health professionals also conveyed that CR should not be articulated as an option or solely as an exercise programme. This downplays the importance of CR and if they did so, the patients therefore would not necessarily see the need to prioritize CR.

“*My experience is that when you say to them [the patients] that CR is not solely exercise, it is actually part of your treatment, this can be an eye-opener for some of them.” (physiotherapist)*.

As the quotes illustrates, the health professionals identified some existing barriers to CR that were related to the articulation of CR.

Some patients do not show up to CR or cancel their appointments in the primary health settings (15). In the health professional's experience, some patient's adherence is easier to achieve in a hospital as some patients find it more important to adhere to their appointments when they are located at a hospital rather than within primary health settings.

“*I think there is a general attitude towards it being easier if you get a letter from the hospital; then it is sometimes easier to show up compared with the primary health settings.” (physiotherapist)*.

From the health professional's point of view, rehabilitation should be initiated early in the patient's treatment and everyone involved should have insight into CR and address it as an important part of the treatment. This may potentially help patients in the transfer from the hospital to the primary health setting and ensure that patients experience a coherent course of treatment and rehabilitation. And help facilitate adherence to CR in the primary health setting.

### Flow of Information

Being aware of the flow of information among everyone involved in the rehabilitation process might facilitate adherence to CR. The health professionals emphasized that sharing more than clinical information about a patient might be one way of facilitating adherence.

“*This man is diagnosed with anxiety and depression so the hospital must have taken this into consideration, but how did they collaborate with him? This information would be beneficial for us to have.” (dietitian)*.

As the quote illustrates, sharing information about patients may facilitate a smoother transition and enhance the process. The health professionals also expressed that for non-native Danish speaking patients who need an interpreter, simple information about what languages they speak would help them in their initial contact. Thus, learning from each other and sharing more than clinical details about patients was one facilitator highlighted in this study.

Work-related environmental factors such as work tasks, terms of employment and working conditions also interact with the patient's overall health and are important factors in rehabilitation (20). This was also a focus for the health professionals in this study, emphasizing the need for information for employers.

“*Giving the employer information regarding CR can possibly make them more flexible or make them realise that CR is an important part of the treatment.” (nurse)*.

A rehabilitation process is complex and involves several different contributors. Ensuring the flow of all relevant items of information between health professionals involved is one way of improving patient adherence to CR in the primary health setting according to the health professionals.

### Contextual Factors

As demonstrated in a previous, related study, comorbidities act as a barrier to adherence to CR (15). Health professionals point out the importance of other contextual factors, including the patient's family and job situation.

“*Having comorbidities and recurring hospitalisation are frequently seen, but also, we have had some patients with a spouse with dementia … We see non-adherence because the everyday life is quite different for those patients, even though they have been referred for CR, we see a different picture and that is something what makes it quite difficult.” (nurse)*.

As the quote illustrates, dropout is not always due to lack of motivation or commitment on the part of the patient. Rather, dropout may be influenced by challenges encountered in everyday life. According to health professionals, facilitating adherence to CR for these patients may be difficult as some of these contextual factors are non-modifiable by the health professionals.

Other contextual factors such as a lack of flexible hours was a barrier identified in a study (15). This was also identified by the health professionals.

“*We have these weight loss programmes and we have had success by changing the time so that instead of an afternoon class from 2 to 4 PM, we now teach the class from 4 to 6.30 PM and that alone allows them to attend, especially those who are still working … I know it is a bit late, but if it is possible to do this in CR, I think it may help adherence.” (dietitian)*.

Facilitating adherence to CR goes beyond simply motivating patients. Other factors may be influencing their decision not to attend. As previously stated, health is more than absence of disease and many different factors influence a patient's health. Thus, health professionals highlighted the importance of including these factors in their work (21).

### Feeling of Belonging

The health professionals made an effort to establish a feeling of belonging for patients in relation to the health professionals and the other patients. From their perspectives, a feeling of belonging may occasionally be essential in the patient's choice to participate in CR.

“*For those who says no to CR, I am putting a lot of effort into creating a relationship with them so that maybe they'll end up saying: Okay, I can come and have a talk with you, because you aren't so bad after all.” (nurse)*.

They highlighted that patients who were difficult to get in to contact with might also be difficult to motivate for participation. In these cases, the initial contact is crucial. In the health professionals' perspectives, the feeling of belonging may trigger reflections and allow for exchange of experiences.

“*Hearing from somebody else; “Okay, you also felt pain when you started, but you are still here. Nice. Well, then I'm not special.” I think it helps a lot that they have each other to talk to. We health professionals can talk all day long, but hearing it from someone who's in the same boat really means a lot.” (physiotherapist)*.

The quote illustrates that in the health professional's view, it was beneficial for patients to share experiences and connect with other patients who were experiencing situations similar to their own, and it was a considerable part of their work to help facilitate this.

## Discussion

The overarching finding from the focus group interviews was that although the health professionals already focused on facilitating adherence, there was room for improvement. During the focus group interviews, the health professionals therefore also shared experiences and ideas about how to address patients who wanted to drop out from CR.

No “one size fits all” exists when it comes to adherence. Patients have different preferences, objectives and life circumstances. Thus, different needs are to be accommodated and addressed in group-based rehabilitation, the health professionals explained. Bringing the person to the center of CR and being able to make adjustments to accommodate patient needs were highlighted as important. Being aware of factors in the patient's daily life was also mentioned as important as these factors influence patient's ability and motivation toward CR. Furthermore, the panel highlighted the importance of placing the person at the center of CR, ensuring that they felt being heard and seen as individuals. Additionally, studies highlights the need for health professionals to be able to make adjustments to suit individual participant's needs and consider the patient's social context including his or her belief toward illness (22, 23). A study from 2021 investigating how nurses responded when faced with patients who were reluctant toward CR reported similar results (24). The nurses in the study also made individual assessments of how and how much to motivate different patients. Highlighting benefits of CR, not being patronizing toward patients and exploring the individual motivations were some of the strategies voiced. Furthermore, respecting patient's autonomy and their right to decline participation was also an important factor (24). Health professionals in the focus groups also underlined the importance of these factors.

According to the focus groups, assuring programme coherence when transferring patients from a hospital to primary health settings was an important factor in facilitating adherence. The panel also stressed the need that health professionals at hospitals had insight into the contents of CR in the primary health settings and knowledge of what patient might expect from CR. Existing guidelines are designed to ensure consistency and coherency in the treatment and rehabilitation of patients with cardiac disease (10). However, according to the health professionals, ensuring a coherent programme requires consistency in communication and dissemination of information about CR. More specifically, CR should be addressed as an important part of their treatment. Furthermore, CR should be implemented in the early stages of treatment and not be limited to the period after discharge. A study found that 45% of patients reported receiving information about CR from the physiotherapists, and 20% reported that a doctor had mentioned CR. When asked about the contents of CR, 27% described CR as an exercise programme exclusively (25). This further stresses the need for a more formalized and rigorous approach to and communication about CR throughout the treatment course.

Flow of information between settings may be improved as reported in the focus groups. Simple information about comorbidities, psychological conditions and language difficulties were the primary information items mentioned. Being equipped with this information prior to their initial meeting with patients may help professionals improve meeting outcomes and thereby facilitate CR adherence. Studies also show that patient outcomes, safety and satisfaction are associated with a close collaboration and coordinated communication between health professionals within rehabilitation services (23, 26). Focus on communication between settings and a more detailed flow of information may yield a smoother transition. Rehabilitation is a biopsychosocial approach aiming to optimize patient's functioning through interventions aimed at both biological health and lived health in context (27). The International Classification of Functioning, Disability and Health classifies the experience of living with a health condition with a focus on physical, mental and social functioning in relation to personal and environmental factors (28). As illustrated in this study the health professionals also emphasized the need for information for employers. A related study showed that challenges with one's employer is a reason why some patients drop out from CR. Hence, for patients who are still of working age, informing of their condition and entering into a dialogue with their employer are important elements (15).

Comorbidities are common among patients with cardiac disease (29). In the health professional's experience, comorbidities were a recurring issue and should be a focus area when addressing any patient. However, the patient's own health was not always the only drop-out reason. Spouses having other diseases such as dementia posed a challenge. A study has shown that living with a spouse with dementia can be challenging and change family dynamics, i.e., some may have to take responsibility for more household chores and stay more at home (30). Thus, living with a severely ill spouse also constituted a barrier to attending CR.

Feeling of belonging is an important facilitator of adherence according to the health professionals. Interaction between the health professionals and the patients is important for the patients to feel welcome and safe. Furthermore, relationships between the participants facilitate sharing of knowledge and experiences and provide a forum where current issues may be discussed with peers. This was also confirmed by the panel. In their experience, patients gained much from talking to others who were facing a similar life situation; whether it was returning to work, being unemployed or retired. From their perspectives, this is the most important factor to incorporate when health professionals compose rehabilitation groups. Patients also found it motivating when the relationship with the health professionals was based on trust. A study exploring patient's experience with an inpatient stay also found that the social relations and interactions were valued by patients (31). The opportunity to exchange experiences and accomplishments with peers throughout their rehabilitation process was reported as a motivational factor that enhanced patient's hope and joy. Furthermore, a feeling of belonging is vital for patient's quality of life and for the benefits that a rehabilitation programme may produce (31). To facilitate equal opportunities for all patients, health professionals should therefore focus on facilitating a feeling of belonging and be aware of different patients' needs and abilities when entering into social relations.

One strength in this study was the broad variation of health professionals. All professions working with CR in the primary health settings were represented in the focus groups. This facilitated a dynamic and diverse discussion covering a wide range of CR aspects. Another strength was the inclusion of panel member perspectives, which may have enhanced the relevance to practice and further validated the results.

However, representatives from only five primary health services were included in the focus groups. Even though these primary health services represented a total of 635,000 inhabitants and were varied with respect to population size, population density and mix of urban and rural areas, having included other primary health settings might have produced more varied results. Furthermore, the interview guide was developed based on results from a previous study exploring patients causes for dropout (15). Even though the focus group discussions were semi-structured and participants were invited to bring forward anything they saw as relevant, the interview guide did guide the discussion. Therefore, the health professionals might have had other perspectives that were not voiced.

In conclusion, the findings of this study provide insight into health professional's perspectives on how to facilitate CR adherence in a primary health setting. Barriers to CR are not generic and transferable from one patient to another. Hence, placing the person at the center of CR is key. This finding is not restricted to somatic and clinical aspects of the patient, but includes their social context, culture and life circumstances. Perceiving these elements as potential facilitators and barriers is of vital importance. Furthermore, the rhetoric related to CR may influence adherence. Adopting a generic and collective approach to and perception of CR throughout the entire course of treatment may help patients comprehend the importance of CR. Further studies are needed to explore patient's perspectives on CR dropout in primary health settings as these perspectives may vary from the health professional's perspectives.

## Data Availability Statement

The original contributions presented in the study are included in the article/Supplementary Material, further inquiries can be directed to the corresponding author.

## Ethics Statement

The studies involving human participants were reviewed and approved by Danish Patient Safety Authority (ID: 31-1522-28). The patients/participants provided their written informed consent to participate in this study.

## Author Contributions

MR drafted the manuscript. All authors revised the manuscript critically, gave their final approval and agreed to being accountable for all aspects of the work thereby ensuring its integrity and accuracy, and contributed to designing the study and data analysis.

## Funding

This project was funded by Public Health in the Central Denmark Region-a joint effort counting both municipalities and the Region, Grant No. A2617.

## Conflict of Interest

The authors declare that the research was conducted in the absence of any commercial or financial relationships that could be construed as a potential conflict of interest.

## Publisher's Note

All claims expressed in this article are solely those of the authors and do not necessarily represent those of their affiliated organizations, or those of the publisher, the editors and the reviewers. Any product that may be evaluated in this article, or claim that may be made by its manufacturer, is not guaranteed or endorsed by the publisher.
