# Supplementary material for: How to Facilitate Adherence to Cardiac Rehabilitation in Primary Health Settings for Ischaemic Heart Disease Patients. The Perspectives of Health Professionals
Source: Front Rehabil Sci. 2022 Mar 28;3:837175. doi: 10.3389/fresc.2022.837175 (PMC9397980; doi:10.3389/fresc.2022.837175)
Supplement: Supplementary file 1 [file Table_1.DOCX]

Supplementary Material

# Case presentations used for focus group discussions

**Case 1**

Rachel is a 68-year-old woman diagnosed with acute myocardial infarction. She is a former nurse and used to work with cardiac patients for many years. She has an adult daughter and three grandchildren and is living with her husband in a small house with a garden. She is normally relatively active and participates in a group coined "Nordic walkers" where she and four other women walk with walking sticks. Furthermore, she rides her bike to and from all activities and feels that this is helping her to keep more or less in shape.

She has participated in: the initial meeting, patient education: 1, physical training: 0, final meeting: 0.

Rachel cancels the first training session and informs us that, due to her educational background and previous work, she feels equipped to manage the training herself. She also feels that she is equipped to understand her body’s signals. She therefore does not wish to participate in CR.

**Case 2**

Chris is a 59-year-old man diagnosed with angina pectoris. He is active and participates in various sports and feels younger than his age. He has two adult children, is divorced and lives alone. He has a house with a garden and enjoys relax doing gardening in his spare time.

He has participated in: the initial meeting, patient education: 2, physical training: 4, final meeting: 0.

Chris feels fit and no longer feels any discomfort due to his cardiac disease. He has resumed both golf and riding his mountain bike and is very motivated to regain his former fitness level. He cancels several training sessions and patient education. He no longer wishes to participate in CR and wants to handle training on his own. He does not feel that the training sessions are at a sufficiently high level. He prefers hiring a personal trainer where he feels he may be pushed so that he may sooner regain his former fitness level.

**Case 3**

Leon is a 46-year-old man diagnosed with acute myocardial infarction. His fitness level is very low, he is a smoker and has experienced increasing dyspnoea for several years. He has two young children but lives alone in a small apartment. Due to his living situation and health condition, he does not see his children very often. He is motivated for participating in CR but has some psychological challenges. He suffers from anxiety and depression and does not feel comfortable in large groups.

He has participated in: the initial meeting, patient education: 2, physical training: 1, final meeting: 0.

At the initial meeting, Leon enquires with great curiosity about the number of people who will be present at the training sessions and how long the sessions last. He initiates CR despite his initial concerns. He participates in the first training session with no physical discomfort. However, he finds it strenuous. He does not show up for the following training sessions or for patient education. He finally sends a text message explaining that his anxiety and depression have increased and that he is unable to follow through with CR.

**Case 4**

Martin is a 55-year-old man diagnosed with ischaemic heart disease. He does not experience any discomfort relating to his condition and describes his general state of health as good. He has never been married and does not have any children. He lives in a houseshare with peers. They arrange communal eating a few times a week and do a lot of different activities together. He feels that they are like a family to him and he also has close friends.

He has participated in: the initial meeting, patient education: 1, physical training: 0, final meeting: 0.

Martin is very motivated to participate in CR and wants to regain his former level of functioning. Even so, he fails to show up to the first training session and later explains that he is having trouble balancing CR and work. He wants to prioritise CR, but feels that his employer is concerned about him being absent. He feels that his employer will frown on him for participating in CR even though he only "requested" flexibility in relation to his work. He does not show up to the fitness test or training and eventually chooses to exercise on his own to avoid workplace conflicts.
